# Does cultural context matter? Comparison of Finnish and Italian children’s social-pragmatic comprehension

**DOI:** 10.3389/fpsyg.2026.1856645

**Published:** 2026-09-04

**Authors:** Ilaria Gabbatore, Eeva Leinonen, Leena Mäkinen, Francesca Marina Bosco, Soile Loukusa

**Affiliations:** 1 Department of Humanities, University of Turin, Turin, Italy; 2 GIPSI Research Group, University of Turin, Turin, Italy; 3 Kathleen Lonsdale Institute for Human Health Research, Maynooth University, Maynooth, Ireland; 4 Research Unit of Logopedics, Faculty of Humanities, University of Oulu, Oulu, Finland; 5 HLS-Fondo Oy, Oulu, Finland; 6 Department of Psychology, University of Turin, Turin, Italy; 7 Finnish Association of Speech and Language Therapist, Helsinki, Finland; 8 ComParte Oy, Oulu, Finland

**Keywords:** assessment, children, cultural factors, language development, pragmatic comprehension

## Abstract

**Introduction:**

While social-pragmatic comprehension has been studied from a number of perspectives, there are only a few studies that focus on cultural factors and their effect on the development of pragmatic processing skills. Previous studies have suggested that various communicative features and different communicative styles may be detected in individuals coming from different contextual backgrounds, even within Europe itself.

**Methods:**

In this light, the present study compared the performance of 150 Finnish and 150 Italian children matched for age and gender (age range 4–8 years) on the Pragma Test, a tool that measures social-pragmatic comprehension using structured protocols.

**Results:**

Results showed a similar developmental trend in pragmatic comprehension among the two groups, while data suggest the Finnish children performed slightly better across all the age groups, even if at age four and five the difference was less pronounced. A difference in performance emerged at age six and persisted until age eight. Italian and Finnish children performed similarly on questions assessing children’s recognition of feelings in context. Differences were noted in questions on contextual processing not involving theory of mind and on understanding the relevant use of language. Finnish children slightly outperformed Italian children in explaining their correct answers.

**Discussion:**

This study enhances our understanding of the development of pragmatic comprehension from a cross-cultural perspective. It highlights universal underpinnings in pragmatic processing as well as some cultural influences that bear upon contextual processing.

## Introduction

Social-pragmatic comprehension develops through childhood and particularly during pre-school and first few years of schooling. Several researchers (see Matthews et al., 2018 for a review) have investigated pragmatic development from a number of perspectives ranging from a focus on development of narrative skills, to the use of speech acts and indirect language (Bosco and Gabbatore, 2017a; Bosco and Gabbatore, 2017b; Bosco et al., 2013; Mäkinen et al., 2018; Pronina et al., 2023; Tonini et al., 2023). However, very few studies (see, e.g., Fukushima and Sifianou, 2017; Gabbatore et al., 2023a; Huttunen et al., 2013; Iverson et al., 2008; Mäkinen et al., 2020) have focused on the effect of cultural factors in the development of pragmatic processing skills. Indeed, given how languages encode meaning distinctions differently and how cultures organize social interactions to emphasize relevant meanings, comparative studies are crucial for understanding pragmatic development (Küntay et al., 2014).

The use of language and other expressive means in a particular context for a communicative effect corresponds to pragmatic ability (Bara, 2010; Holler and Levinson, 2019; Levinson, 1983). Context can be referred to as a multidimensional concept including cognitive, social, linguistic, and non-linguistic (i.e., prosody, gestures and bodily communication) components, cultural aspects, as well as the physical environment (Gibbs and Colston, 2012; Prutting, 1982; Sperber and Wilson, 1995). Communicating in a particular context implies also the ability to direct attention to relevant contextual factors, to engage in inferencing and to understand other people’s intentions, emotions and motives (Sperber and Wilson, 2012). When interpreting language in context, inferential ability allows one to connect a multitude of contextual information that is available for the listener (Bosco et al., 2004; Leinonen et al., 2000). Beyond successful comprehension itself, pragmatic processing may also involve awareness of the contextual and inferential factors supporting one’s interpretation. In other words, children may arrive at an appropriate pragmatic interpretation without necessarily being able to explicitly identify or verbalize the cues and reasoning underlying their understanding (Donaldson, 1986; Letts and Leinonen, 2001). This ability to reflect on and articulate the basis of one’s own understanding can be viewed as a form of metacognitive awareness related to pragmatic processing.

Contextual comprehension is also shaped by personal perspectives and expectations (O’Neill, 2012) and relates to the ability to take into account one’s own and others’ mental states, such as beliefs and intentions and act accordingly, which is referred to as Theory of Mind (ToM; Premack and Woodruff, 1978).

The abilities required for the processing of contextual information develop between the ages of four and eight, which is evident in children’s increased ability to understand more sophisticated meaning in communication (e.g., Bosco and Gabbatore, 2017b; Bosco et al., 2013; Falkum and Köder, 2020; Gabbatore et al., 2025a; Helland et al., 2024; Loukusa and Leinonen, 2008; Loukusa et al., 2007). At the same time, cognitive and social abilities, including ToM, develop through childhood (Saxe, 2013; Wellman and Liu, 2004) and adolescence (Bosco et al., 2014; Bosco et al., 2016; Brizio et al., 2015).

Moreover, some previous evidence suggests that children’s gender may somehow impact the developmental patterns of communicative abilities. Girls often outperform boys in several linguistic aspects, such as engaging more easily in spontaneous conversations (e.g., Bauer et al., 2002), and demonstrating superior storytelling skills (Fey et al., 2004). Recent findings also indicate that girls may slightly outperform boys in contextual and social comprehension abilities (Helland et al., 2024). However, other studies failed to detect any gender-related difference in communication or social skills (e.g., Collins et al., 2014; Helland et al., 2009; Law et al., 2014; see also Etchell et al., 2018 for a review), as well as in metacognitive ability (e.g., Xue et al., 2024). Therefore, taking a closer look at this variable could provide useful insights.

Also, children’s communication style might also be influenced by a complex interplay of variables, including the school system, the societal structure, the organization of children’s free time and leisure activities, as well as family household and management, such as parents’ working hours and availability, or the presence of siblings, which all together contribute to the construct of socioeconomic background. An influence of such factors was found on children’s language development, as for example vocabulary or syntax (see Rowe, 2018), while less is known on the extent to which SES affects children’s pragmatic performance (see Schulze and Saalbach, 2022 for a recent analysis of the variety of results on this topic).

Determining children’s ability to engage in pragmatic comprehension allows caregivers and professionals to better understand and support children in everyday communicative situations. In this endeavor it is increasingly important to consider cultural and linguistic factors. Comparing pragmatic development and performance across different languages and cultures has not been often undertaken because of a lack of common assessment protocols for pragmatic performance (see Gabbatore et al., 2019). However, studies exist that have focused on how regulation of communication style can vary across cultures and languages (Matsumoto and Hwang, 2016). This variation can be seen in the way individuals express requests (Cohen and Olshtain, 1993), complaints (Boxer, 1993), and refusals (Kwon, 2004), as well as how politeness is intended and expressed (see Küntay et al., 2014, see also Ogiermann, 2009). Cultural differences have also been observed in the use of an appropriate amount of speech and eye contact, and how turn-taking and other communicative devices are used (Payne and Taylor, 2002; see also Küntay et al., 2014).

The above-mentioned variability appears to be detectable also within the European context. Although EU member states share an increasingly unified cultural framework through their association with the European Union, each country retains distinct cultural and communicative characteristics. For instance, the Finnish communication style is often stereotyped as quiet, reserved, introverted, and taciturn (Lehtonen and Sajavaara, 1985; Sallinen-Kuparinen et al., 1991), and Finns are known to be quite comfortable with extended silences during conversations (Sallinen-Kuparinen, 1986). In contrast, Italians are generally perceived as more talkative and less tolerant of silence between conversational turns, often interpreting silence as a sign of indifference, opposition, or distrust (Agliati et al., 2005). Moreover, Finnish communication shows a less marked distinction between formal and informal speech, particularly in the use of requests and expressions of courtesy (see Gabbatore et al., 2019), as well as in the way politeness markers are conveyed (Peterson and Vaattovaara, 2014). Italian communication, on the other hand, is characterized by the frequent use of culturally specific representational gestures during interactions (e.g., Kendon, 2004a). Cultural differences were found in narrative productivity with children from the Mediterranean area, i.e., Italian, being more talkative than children from Nordic countries, i.e., Finnish and Canadian, as early as age four (Mäkinen et al., 2020). In a similar cross-cultural frame, Huttunen et al. (2013) found that the British children used more gestures as compared to Finnish children in a picture naming task. Different communication styles may be culturally learned (Jokinen and Wilcock, 2006) and children’s pragmatic development may be influenced by different linguistic and cultural contexts. Gabbatore et al. (2023a) performed a comparison of the communicative performance of Finnish and Italian typically developing children as assessed by parents using the Children’s Communication Checklist–2 (CCC-2; Bishop, 2003). The authors found an effect of nationality on most subscales, with Finnish parents rating their children’s communicative abilities higher than Italian parents; a pattern of differences and similarities that should be considered when conducting communicative assessments. The results of the study were discussed on the basis of cultural characteristics that shape communicative style within societies, but also taking into account the fact that CCC-2 includes the cultural perspectives of the parents themselves, which may have influenced the results. Therefore, the few cross-cultural data available seem to suggest that pragmatic performance needs to be assessed from different perspectives, considering both parents’ reports and children’s actual performance in experimental situations. In the present study, we directly investigate and compare the performance of Finnish and Italian children in an experimental setting.

### The present study

While there are a number of studies conducted regarding the development of social-pragmatic understanding (e.g., Bosco and Gabbatore, 2017b; Bosco et al., 2013; Del Sette et al., 2021; Falkum and Köder, 2020; Gabbatore et al., 2025a; Loukusa and Leinonen, 2008; Loukusa et al., 2007), there is a need for systematic research with samples of age- and gender-matched children from different linguistic and cultural backgrounds, in order to better understand the effect of different contextual factors in the development of pragmatic comprehension. In this study, we compare children’s performance on the Pragma Test (Loukusa, 2019) in two different cultural and linguistic contexts. Please note that we use the term *cultural context* to refer to the broad developmental environment in which children acquire communicative competence. This environment encompasses multiple, partially overlapping dimensions, including the language spoken by the child, culturally shared communicative practices, educational experiences, family interaction patterns, and broader socioeconomic conditions. These dimensions are very likely to interact throughout development and cannot be considered independent contributors to pragmatic competence. Consequently, nationality – Finnish and Italian in this specific case - is not meant to be interpreted as a causal variable per se, but as an indicator of different developmental contexts characterized by a constellation of linguistic, educational, familial, and sociocultural experiences.

The pragmatic assessment tool adopted within the present study was developed in Finland (Loukusa et al., 2017) and translated and adapted to Italian (Gabbatore et al., 2021). This gives us a unique opportunity to compare, in a structured situation using the same methodology, the pragmatic development of children belonging to two different linguistic and cultural contexts. No previous study has examined children’s social-pragmatic comprehension abilities or profiles cross-culturally using the Pragma Test, nor directly compared age- and gender-matched Italian and Finnish children’s social-pragmatic comprehension.

We compare Finnish and Italian children’s performance on the Pragma Test as a function of age, both considering total scores and the scores on the five different question types comprising the Pragma test. On the basis of earlier studies (e.g., Bosco et al., 2013; Helland et al., 2024; Loukusa et al., 2017; Ryder and Leinonen, 2003) we know that children’s performance in pragmatic tasks is affected by the level of inferential and contextual complexity, but we do not know precisely whether cultural or language background has an effect on the pattern of performance as assessed by the Pragma Test.

HP1: In line with results of previous works using the CCC-2 (Gabbatore et al., 2023a), we expect similar developmental trends in the two cultural groups. However, we also expect to observe slightly higher scores for Finnish children in line with the results of previous evidence (see Gabbatore et al., 2021; Loukusa et al., 2017) which used the Pragma test in different samples of Finnish and Italian preschool- and school-aged children without directly comparing them. Based on the previous data available, the slight expected Finnish advantage may be more pronounced for some pragmatic tasks (e.g., contextual inferences) than for others (i.e., feeling recognition) and it may vary across development, being less evident in the early years and becoming more pronounced as children grow older.

Furthermore, based on the earlier research (e.g., Letts and Leinonen, 2001; Loukusa et al., 2008) we know that children’s ability to explain their correct answers develops between the ages of four and eight. However, to our knowledge there are no earlier studies comparing explanatory abilities of children cross-culturally. Understanding how different contextual environments may or may not influence pragmatic development provides insights into how specific factors such as culture and language may influence children’s performance along pragmatic development (Gabbatore et al., 2023a; Mäkinen et al., 2020; Peña, 2007). A deeper understanding of the effect of cultural factors on social-pragmatic comprehension is also important when identifying communication difficulties in clinical populations (see, e.g., Dindar et al., 2023; Gabbatore et al., 2023b; Gabbatore et al., 2025b; Gabbatore et al., 2022; Guerrini et al., 2026; Tuohimaa et al., 2022) as these difficulties affect everyday communication and relationships with others along development and beyond. In addition to successful pragmatic comprehension, the Pragma Test also examines children’s ability to explicitly explain how they arrived at a correct interpretation. These explanation tasks require children not only to have inferred the intended meaning successfully, but also to consciously identify and verbalize the contextual, social, or intentional cues that supported their interpretation. As such, they provide an indirect measure of children’s metacognitive ability, namely the ability to reflect upon and explain the pragmatic processes underlying successful communication (Loukusa et al., 2008, 2017). To our knowledge, no previous study has examined the development of such ability from a cross-cultural perspective. Considering both successful pragmatic comprehension and children’s ability to explicitly identify the basis of their interpretations may provide a broader picture of pragmatic development than comprehension accuracy alone.

HP2. We expect Finnish and Italian children to show similar developmental trajectories in metacognitive awareness, as reflected by their performance on the explanation tasks, in line with previous developmental evidence obtained separately in Finnish and Italian samples (Gabbatore et al., 2021; Loukusa et al., 2017). A broadly similar developmental trend has also been observed in Norwegian children (Helland et al., 2024), further suggesting the expected cross-linguistic consistency in metacognitive development.

Finally, while some previous studies identified some gender-based difference in conversation management with girls outperforming boys (e.g., Bauer et al., 2002), some others did not detect any effect of gender in, for example, communication or social skills (e.g., Collins et al., 2014). As the literature on this topic still suggests controversial results (e.g., see Etchell et al., 2018 for a review), we examine, for explorative purposes, the possibility to detect gender differences along the development of both pragmatic comprehension and metacognitive awareness, in the two cultural contexts compared in the present study. Specifically (HP3), in line with previous comparisons on pragmatic ability performed with other assessment tools (see Gabbatore et al., 2023a), we expect girls to outperform boys with a nuance of results at the different pragmatic tasks that may vary as a function of age and nationality.

## Methods

### Participants

A total of 300 children (150 Finnish and 150 Italian), of which there were 74 girls and 76 boys in both groups, participated in the study. The children’s ages ranged from 4 to 8 years and were divided into five age groups (see Table 1). The Italian and Finnish samples were totally comparable in terms of age (*t* = 0.33; *p* = 0.742; *Cohen’s d* = 0.04).

**Table 1:** Participants’ demographic characteristics.

Age group	*N*		Age (years; months) mean (SD)	
	Ita	Fin	Ita	Fin
A (4–4;11)	30	30	4;5 (0;3)	4;6 (0;3)
B (5–5;11)	30	30	5;4 (0;2)	5;5 (0;3)
C (6–6;11)	30	30	6;6 (0;2)	6;6 (0;2)
D (7–7;11)	30	30	7;5 (0;3)	7;8 (0;2)
E (8–8;11)	30	30	8;5 (0;3)	8;4 (0;3)
Total	150	150	6;5 (1;5)	6;5 (1;4)

The children’s language and cognitive development was explored via a preliminary questionnaire filled in by the children’s parents. The questionnaire contained questions about the child’s early language development (e.g., *At what age did the child start using intelligible words*?) and was administered as a screening tool to verify the inclusion and exclusion criteria (e.g., developmental milestones, hearing, previous therapies, home language, and familial risk) rather than as a standardized developmental assessment. All the children included in the analyses of the present study, in both the Finnish and the Italian sample, did present developmental milestones within the normative range (e.g., first word around 1 year, intelligible speech at 3 years of age, absence of neurodevelopmental disorder). A description of the questionnaire is reported in Table 2.

**Table 2:** Brief description of the screening questionnaire filled by the parents.

Domain	Variable collected	Response format	Purpose
Family history	Family history of developmental language or neurodevelopmental disorders (e.g., dyslexia, delayed language acquisition, hearing impairment, intellectual disability, other developmental disorders)	Yes/No; if yes, degree of kinship and type of disorder	Screening for familial risk factors
Early development	Overall course of early development	Yes/No; if atypical, open-ended description of difficulties	Screening for atypical developmental history
Early language milestones	Age at first word	Open-ended (age)	Assessment of early expressive language development
Early language milestones	Age at first word combinations/sentences	Open-ended (age)	Assessment of early syntactic development
Speech intelligibility	Speech intelligibility at 3 years of age	Yes/No	Parent-reported speech development
Speech-language intervention	History of speech and language therapy	Yes/No; if yes, type of intervention and reason for referral	Identification of previous clinical intervention
Hearing history	Ear infections during the previous year	Yes/No; if yes, number of episodes	Screening for possible hearing-related risk factors
Additional information	Other relevant developmental or medical information	Open-ended	Collection of supplementary information

The participants were recruited via day care centers and primary schools in the north and south of Italy (Piedmont and Campania regions) and in the north and south of Finland (Oulu and Tampere areas). Parents received detailed written information about the study and participation was voluntary and confirmed by written consent by each child’s parent. In Italy, the study was approved by the Bio-ethical Committee of the University of Turin (Protocol no. 23979). In Finland, the study was part of a larger pragmatic communication project which received the statement of approval from the Regional Ethics Committee of Northern Ostrobothnia Hospital District (code: 44/2009).

The children belonged to similar socioeconomic backgrounds, primarily ranging from middle to upper-middle class. This information was gathered from parental occupational data, which served as an index of Status (SES), and was organized according to the International Standard Classification of Occupations (Statistics Finland, 2011): (1) upper-level employees with administrative, managerial, professional or related occupation (e.g., doctor, psychologist, manager, software designer), (2) lower-level employees with administrative or clerical occupation (e.g., nurse, real estate agent, police), (3) manual worker (e.g., postman, hairstylist), (4) self-employed, (5) retired, student or unemployed. The data revealed a similar distribution of occupational levels between the two samples. There was no significant difference in the occupation of Italian and Finnish fathers [χ^2^_(5)_ = 9.31; *p* = 0.095] and some variability in the occupations of the mothers [χ^2^_(5)_ = 11.58, *p* = 0.038], with the Finnish sample being slightly skewed toward higher occupation levels. These data likely reflect the socioeconomic backgrounds of the two countries, with the Finnish mothers’ socioeconomic distribution slightly skewed towards higher occupation levels. In order to verify that this difference did not affect the overall results of the present paper, we conducted a one-way ANOVA with the Pragma total score as the dependent variable and with maternal occupational level as the independent variable. The analysis revealed no effect of maternal occupation on the Pragma total score (*F* = 1.94; *p* = 0.074; η^2^_p_ = 0.036). Post-hoc Tukey corrections showed no significant pairwise differences between any of the occupational categories (0.242 < p_Tukey_ < 1). A similar pattern emerged for the explanation total score: the ANOVA revealed no significant effect of maternal occupation (*F* = 1.78; *p* = 0.10; η^2^_p_ = 0.033), and post-hoc Tukey corrections showed no significant differences between any of the occupational categories (0.221 < p_Tukey_ < 1).

### Materials

The Pragma Test was developed by Loukusa et al. (2017) (see also Loukusa, 2019) for examining how children utilize contextual information, social language use, and the understanding of intentions, thoughts, and feelings in utterance interpretation. The Pragma test was later translated and cross-culturally adapted for use in the Italian context (see Gabbatore et al., 2021), showing good psychometric properties: good levels of content validity, high interrater reliability (intra-class correlation coefficient of 0.985 as for the answer tasks and 0.984 for the explanation tasks), good to excellent internal consistency for both answers (Cronbach’s *α* = 0.927) and explanations (α = 0.794). The adaptation followed a structured translation and back-translation procedure, combined with expert review in both countries and minimal culturally motivated modifications, while preserving the theoretical framework, item structure, administration procedures, and scoring criteria (see Gabbatore et al., 2021). The cross-cultural adaptation was designed to preserve conceptual rather than literal equivalence between the two versions, ensuring that the same pragmatic constructs were assessed while allowing only those linguistic and contextual modifications necessary to maintain the ecological validity of the test in the Italian context.

In both the Finnish and the Italian versions, the test contains 39 questions that require the processing and understanding of meaning which is derived by engaging in contextual processing. The questions explore how children discern meaning by retrieving and integrating contextual information, such as world and social knowledge, the physical context within which the meaning is interpreted and prior verbal information. The Pragma Test consists of short verbal scenarios that are presented with the support of pictures, small plastic characters or animals.

The Pragma Test consists of five types of questions that examine contextual processing.

1 *Contextual inference with ToM demand - ConToM* (*n* = 18)

These questions focus on the child’s ability to discern the intended meaning by connecting information from different sources (e.g., verbally given information, pictorial information and world knowledge) and taking into account others’ mental states and emotions (ToM demand).

2 *Contextual inference without ToM demand* - *ConNoToM* (*n* = 10).

These questions focus on the child’s ability to discern the intended meaning by connecting information from different sources (e.g., information given verbally, pictorial information or world knowledge) but with no requirement to take into account the mental states or emotions of others.

3 *Feelings recognition* (*n* = 5)

These questions focus on the child’s ability to understand feelings based on given contextual cues that are primarily verbal.

4 *Relevant language use – RelUseLang* (*n* = 4)

These questions focus on the understanding of language use relevant to context. This involves choosing to say something in a certain way in order to achieve one’s own goal within a particular situation. This requires utilizing contextual cues as well as social and world knowledge.

5 *False belief* (*n* = 2)

These questions focus on the ability to understand FB tasks (i.e., change-in-location task and unexpected content task). These questions evaluate the ability to understand others’ mental states.

The participants were presented with the 39 questions, and the answers were scored 0 for incorrect and 1 for correct answers. Scores were derived for all 39 items and separately for each question type. The child’s first response was always scored, as this reflected their initial, unassisted performance. Assistance was not provided during the response. If the child explicitly requested that the question be repeated, the examiner repeated it once. In such cases, the score was based on the child’s answer after the repetition. Repetition occurred only when the child asked for it, ensuring that the testing conditions remained standardized. When answers were scored, the question type was checked because it indicated the specific aspect of communication or comprehension that the response was intended to demonstrate (see also Loukusa et al., 2017 for examples of correct and incorrect answers).

Successful pragmatic comprehension does not necessarily imply awareness of the factors supporting that comprehension; therefore, the explanation task was designed to assess children’s metacognitive awareness of the contextual and inferential processes underlying their successful interpretations. The participants were asked to provide an explanation for their correct answers to 13 questions (“How do you know that?”) to explore if they were aware of, and able to articulate, how they had derived the answers based on the contextual information. Because explanations were asked only after correct responses, these scores should be interpreted as reflecting children’s awareness of the basis of successful comprehension rather than a measure entirely independent of comprehension accuracy.

All responses were scored according to the original Pragma administration and scoring manual. The same standardized administration procedures, scoring criteria, and coding manual were used by both the Finnish and Italian research teams. The Finnish responses were scored by researchers from the original Pragma research group, whereas the Italian responses were scored by researchers involved in the Italian adaptation of the test, all of whom received specific training in the administration and coding procedures. Whenever a response was considered ambiguous or difficult to classify, two independent raters discussed the case and referred to the scoring manual until consensus was reached. Because the present study adopted exactly the same administration and scoring procedures as those used in the original Finnish (Loukusa et al., 2017) and Italian validation studies (Gabbatore et al., 2021), both of which demonstrated excellent agreement between independent coders, inter-rater reliability was not recalculated for the present dataset.

### Statistical analysis

In order to compare the performance of the Finnish and Italian children a three-way univariate ANOVA was run, with the Pragma total score as the dependent variable and Nationality, Age group and Gender as the independent variables. In order to compare the scores obtained by the Finnish and Italian children on each of the different types of questions, a multivariate ANOVA was run, with the score for each question type as the dependent variable and Nationality, Age group and Gender as the independent variables.

Furthermore, we examined the children’s performance in terms of their ability to explain their correct answers (metacognitive awareness). Thirteen of the questions contained a follow-up question asking for an *explanation*. As explanation questions were elicited only following correct responses to the inference questions, children’s explanations should be interpreted relative to the number of correctly answered inference questions. Since an explanation was asked for only if a correct answer was given, relative frequency scores were used in the analysis (i.e., the number of correct explanations in proportion to correct answers, i.e., number of correct explanations/number of correct answers × 100%). A three-way univariate ANOVA was run, with the explanation relative frequency score as the dependent variable and Nationality, Age group and Gender as the independent variables.

Effect sizes were reported using partial eta squared (η^2^_p_), with values of 0.01, 0.06, and 0.14 interpreted as small, medium, and large effects, respectively (Richardson, 2011). Bonferroni corrections were applied to account for multiple comparisons and reduce the risk of Type I errors. The analyses were conducted with JASP version 0.95.4.

G*Power 3.1 (Faul et al., 2009) was used to conduct an *a priori* analysis to ensure sufficient power for our analyses, i.e., univariate and multivariate ANOVAs. Statistical power was calculated based on the observed effect sizes in previous studies in which Finnish and Italian communicative performance was compared (Gabbatore et al., 2021, 2023a). The previous evidence indicated a medium effect size (η^2^_p_ = 0.062 corresponding to ƒ = 0.26) for the main effect of age on pragmatic performance. Given an alpha level of 0.05 (2×5 design), G*Power indicated *N* = 280 to get a statistical power of 95%. A similar approach was adopted for interaction effect, for which the previous studies indicated a small-to-medium effect (η^2^_p_ = 0.045 corresponding to ƒ = 0.22); in this case - given the same alpha (0.05) and with a lower power of 85% - for interactions with 1 degree of freedom G*Power indicated that the required sample size was at least 188, while for interactions with 4 degrees of freedom N was expected to be 283. We therefore recruited 300 children to ensure adequate statistical power for all the analyses.

## Results

**Figure 1:**
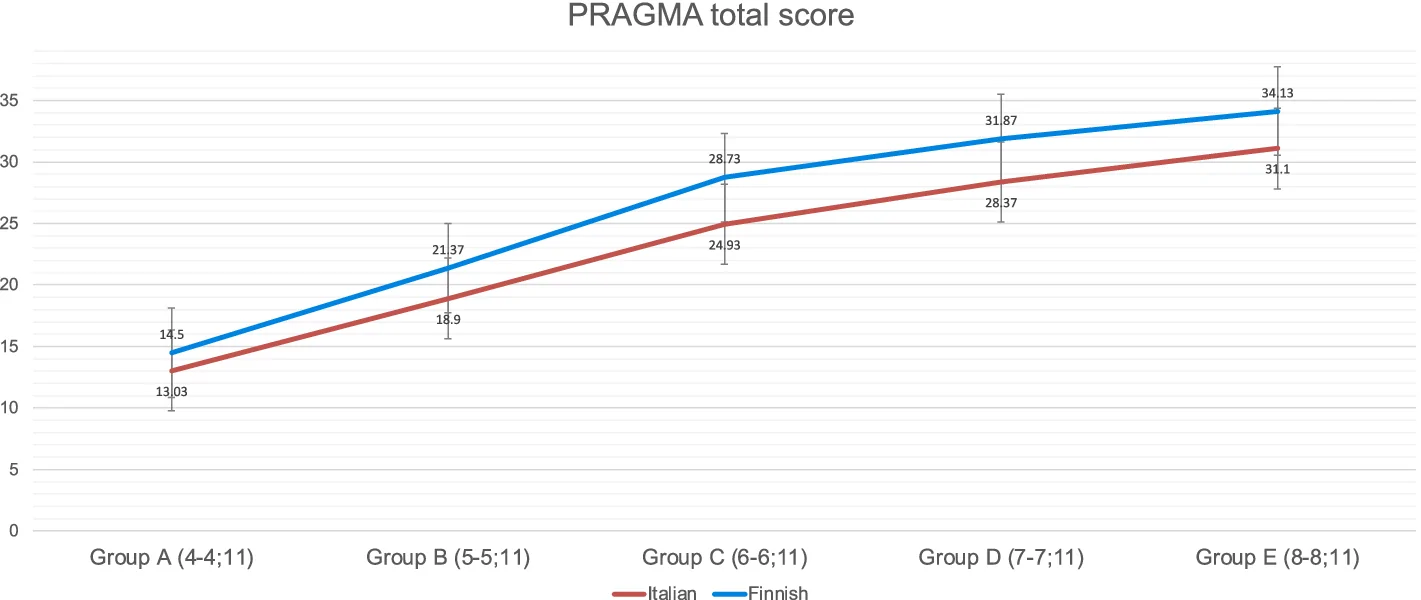
Pragma Test total scores of Finnish and Italian the children across age groups. Data points represent the mean Pragma Test total score for each age group. The blue line refers to Finnish sample and the red line refers to the Italian sample.

The data indicate a similar pattern of performance scores for the Italian and Finnish children, despite Finnish children showing on average higher scores than the Italian children (see Figure 1). The analysis revealed a significant effect of the Age Group [*F*_(4,280)_ = 131.902; *p* < 0.001; η^2^_p_ = 0.653], a significant effect of Nationality [*F*_(1,280)_ = 23.22; *p* < 0.001; η^2^_p_ = 0.076], and a significant effect of Gender [*F*_(1,280)_ = 10.904; *p* = 0.001; η^2^_p_ = 0.037]. The analysis showed no interaction Nationality × Age group [*F*_(4,280)_ = 0.467; *p* = 0.760; η^2^_p_ = 0.007], nor interaction Nationality × Gender [*F*_(1,280)_ = 1.940; *p* = 0.165; η^2^_p_ = 0.007]. The interaction Nationality × Age group × Gender [*F*_(4,280)_ = 1.346; *p* = 0.253; η^2^_p_ = 0.019] was also not significant.

**Figure 2:**
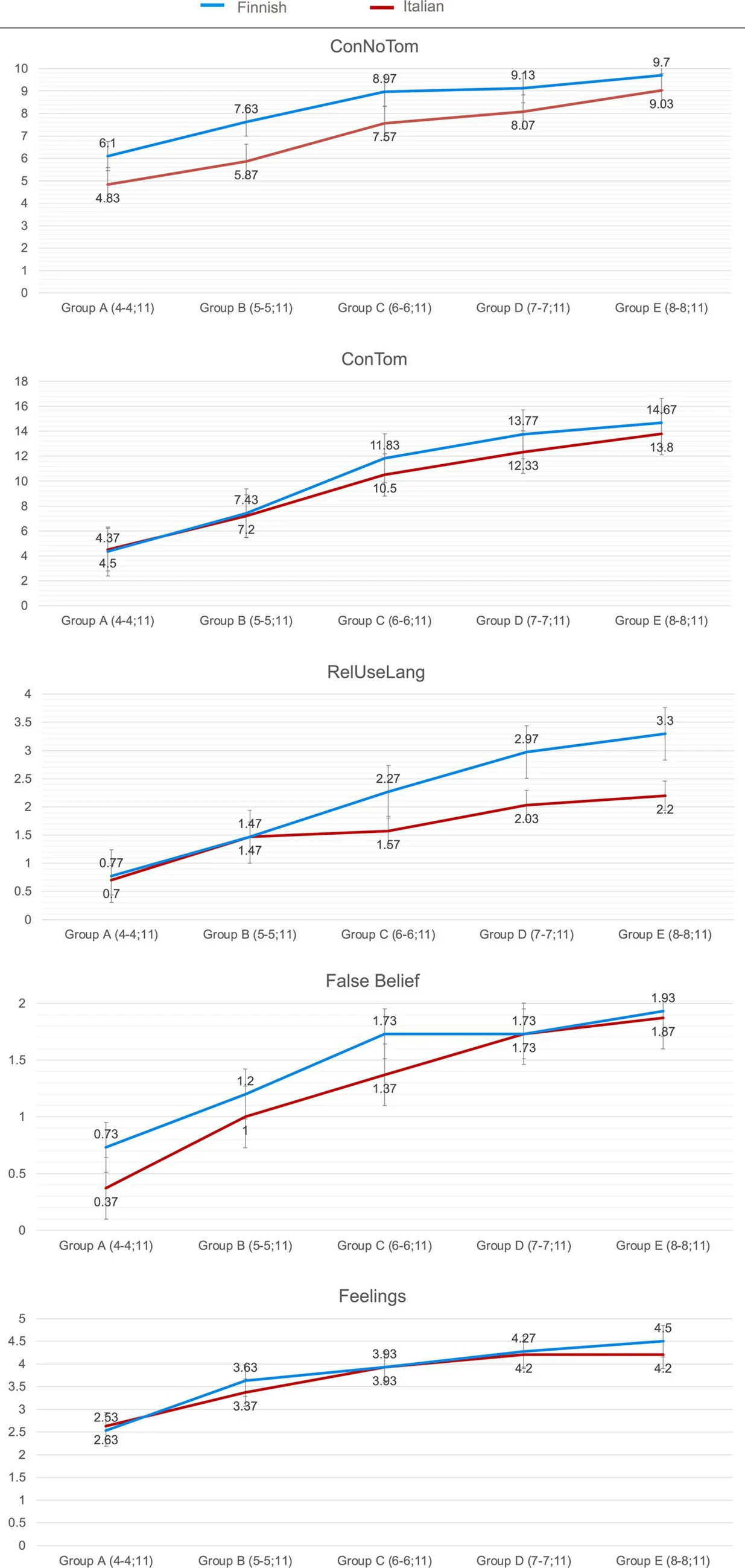
Performance scores of Finnish and Italian children across the different Pragma Test question types. Data points represent the mean scores for each question type within each age group. Blue lines refer to the Finnish sample and red lines refer to the Italian sample.

The results of the multivariate ANOVAs for the different question types are presented in Table 3 and Figure 2.

**Table 3:** Correct answers across Pragma Test question types and comparisons between Finnish and Italian children.

Question type (n. of questions)	Effect of nationality	Effect of age group	Effect of gender	Interaction nationality × age group	Interaction nationality × gender
Contextual inference without ToM demand *ConNoToM* (10)	*F*_(1, 280)_ = 48.87 ***p* < 0.001** η^2^_p_ = 0.150	*F*_(4, 280)_ = 62.96 ***p* < 0.001** η^2^_p_ = 0.473	*F*_(1, 280)_ = 0.14 *p* = 0.706 η^2^_p_ = 0.001	*F*_(4, 280)_ = 1.06 *p* = 0.375 η^2^_p_ = 0.016	*F*_(1, 280)_ = 1.91 *p* = 0.168 η^2^_p_ = 0.007
Contextual inference with ToM demand *ConToM* (18)	*F*_(1, 280)_ = 4.82 ***p* = 0.029** η^2^_p_ = 0.017	*F*_(4, 280)_ = 115.55 ***p* < 0.001** η^2^_p_ = 0.622	*F*_(1, 280)_ = 9.76 ***p* = 0.002** η^2^_p_ = 0.034	*F*_(4, 280)_ = 0.80 *p* = 0.521 η^2^_p_ = 0.011	*F*_(1, 280)_ = 2.131 *p* = 0.145 η^2^_p_ = 0.008
Recognition of feelings *Feelings* (5)	*F*_(1, 280)_ = 1.02 *p* = 0.313 η^2^_p_ = 0.004	*F*_(4, 280)_ = 36.69 ***p* < 0.001** η^2^_p_ = 0.343	*F*_(1, 280)_ = 9.19 ***p* = 0.003** η^2^_p_ = 0.032	*F*_(4, 280)_ = 0.53 *p* = 0.712 η^2^*_p_* = 0.007	*F*_(1, 280)_ = 0.17 *p* = 0.680 η^2^_p_ = 0.001
Relevant use of language *RelUseLang* (4)	*F*_(1, 280)_ = 25.23 ***p* < 0.001** η^2^_p_ = 0.083	*F*_(4, 280)_ = 41.76 ***p* < 0.001** η^2^_p_ = 0.373	*F*_(1, 280)_ = 13.81 ***p* < 0.001** η^2^_p_ = 0.047	*F*_(4, 280)_ = 4.10 ***p* = 0.003** η^2^_p_ = 0.055	*F*_(1, 280)_ = 0.03 *p* = 0.859 η^2^_p_ = 0.001
False belief *FB* (2)	*F*_(1, 280)_ = 7.43 ***p* = 0.007** η^2^_p_ = 0.026	*F*_(4, 280)_ = 44.22 ***p* < 0.001** η^2^_p_ = 0.387	*F*_(1, 280)_ = 2.22 *p* = 0.137 η^2^_p_ = 0.008	*F*_(4, 280)_ = 1.05 *p* = 0.381 η^2^_p_ = 0.015	*F*_(1, 280)_ = 0.16 *p* = 0.690 η^2^_p_ = 0.001

Significant *p* values are reported in bold.

Since the interaction Nationality × Age group was significant for the *RelUseLang* question type (see Table 3), we conducted a *post hoc* Bonferroni comparison to provide a more detailed analysis of the results. The analysis revealed no difference between Finnish and Italian children in group A (4–4;11 years) (*p* = 0.799) and B (5–5;11 years) (*p* = 1.0), while the difference becomes progressively significant in group C (6–6;11 years) (*p* = 0.008), D (7–7;11 years) (*p* < 0.001) and E (8–8;11 years) (*p* < 0.001).

**Figure 3:**
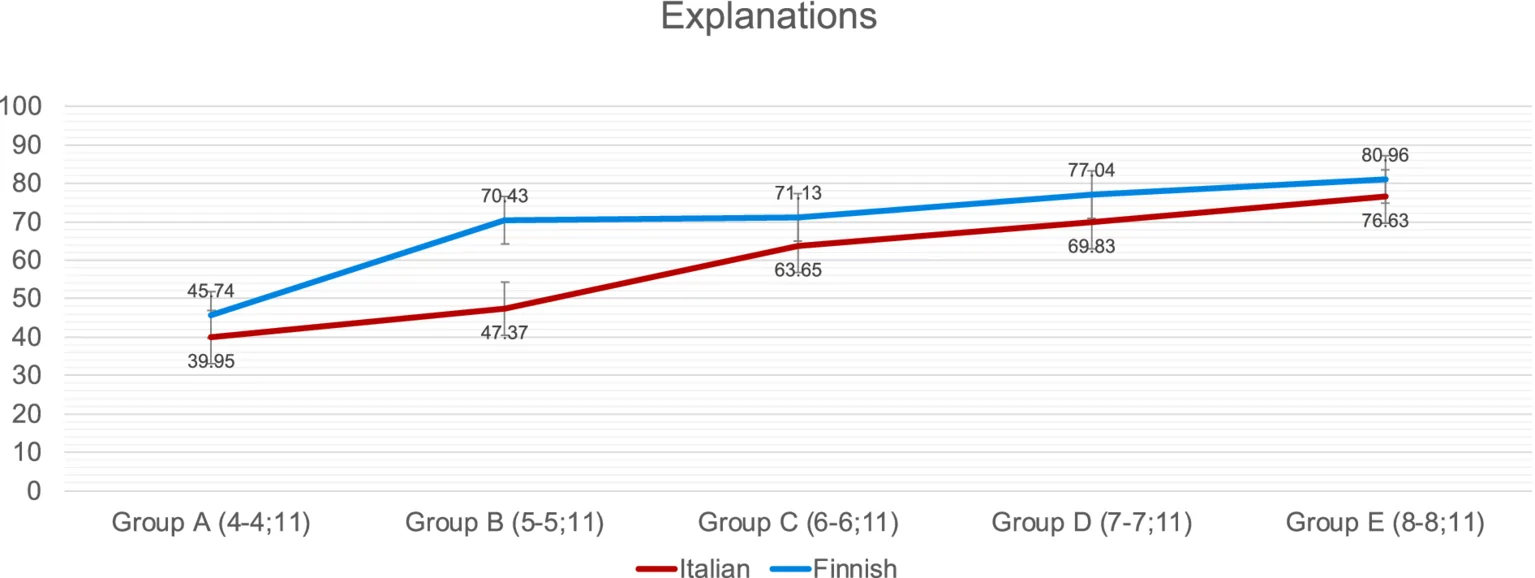
Overall performance scores of Finnish and Italian children on the Pragma Test explanation tasks. Data points indicate the mean scores (relative frequency, expressed in %) for each age group.

As for the explanation tasks of Pragma, reflecting metacognitive awareness, the scores obtained by the Italian and Finnish children were compared using relative frequencies, i.e., number of correct explanations/number of correct answers × 100%. The number of correct explanations for Finnish and Italian children is displayed in Figure 3.

The three-way univariate ANOVA revealed a significant effect of Nationality [*F*_(1,280)_ = 14.61; *p* < 0.001; η^2^_p_ = 0.050], of Age group [*F*_(4,280)_ = 25.04; *p* < 0.001; η^2^_p_ = 0.263], as well as an effect of Gender [*F*_(1,280)_ = 7.11; *p* = 0.008; η^2^_p_ = 0.025]. No interaction Nationality × Age group [*F*_(4,280)_ = 1.87; *p* = 0.116; η^2^_p_ = 0.026] nor Nationality × Gender [*F*_(1,280)_ = 0.05; *p* = 0.818; η^2^_p_ = 0.000] was detected. Overall, Finnish boys got a mean score of 66.07 (SD = 25.06) and Finnish girls an average score of 72.13 (SD = 22.31), while within the Italian sample, boys got a mean score of 55.75 (SD = 27.97) and girls a mean score of 63.33 (SD = 24.23). Mean scores for each group are detailed in Table 4.

**Table 4:** Mean scores obtained by boys (M) and girls (F) on the Pragma Test explanation tasks, by age group and nationality.

Nationality	Age group	Gender	Mean (SD)
Italian	A (4–4;11)	F	44.09 (29.53)
		M	35.82 (32.27)
	B (5–5;11)	F	54.65 (22.81)
		M	41.00 (34.88)
	C (6–6;11)	F	64.75 (20.64)
		M	62.55 (15.40)
	D (7–7;11)	F	75.63 (15.15)
		M	64.04 (14.29)
	E (8–8;11)	F	76.94 (14.73)
		M	76.32 (12.44)
Finnish	A (4–4;11)	F	49.29 (26.63)
		M	42.19 (27.19)
	B (5–5;11)	F	78.85 (14.46)
		M	63.06 (24.94)
	C (6–6;11)	F	71.12 (19.52)
		M	71.14 (23.09)
	D (7–7;11)	F	78.36 (18.17)
		M	75.73 (15.77)
	E (8–8;11)	F	83.48 (14.02)
		M	78.43 (16.27)

### Overall pattern of results

Overall, the results showed a highly consistent developmental pattern across the Italian and Finnish samples. Age emerged as the strongest predictor of pragmatic performance, exerting a significant effect on the overall Pragma score (η^2^_p_ = 0.65), on all question types (η^2^_p_ = 0.343–0.622), and on explanation tasks (η^2^_p_ = 0.263), indicating marked improvements in pragmatic abilities across development.

Nationality had a significant, although substantially smaller, effect on the overall score (η^2^_p_ = 0.076), with Finnish children outperforming Italian children on average. This advantage was specifically observed in a number of question types, i.e., *ConNoToM*, *ConToM*, *RelUseLang*, and *FB*, but not in *Feelings recognition.* Similarly, a between-country difference emerged also for *Explanation tasks*, with Finnish outperforming Italian children.

Gender effects were generally small (η^2^_p_ = 0.025–0.047), with girls outperforming boys on the overall score, *ConToM*, *RelUseLang*, *Feelings recognition*, and *Explanation tasks*, but not on *ConNoToM* or *FB*.

Importantly, almost all interactions involving nationality were non-significant, indicating that the developmental trajectories were remarkably similar in the two countries. The only exception concerned the *RelUseLang* subscale, for which Finnish children began to outperform Italian children from 6 years of age onwards.

## Discussion

The current study compared the social-pragmatic comprehension skills of Finnish and Italian children, matched for age and gender. As previously highlighted (e.g., Gabbatore et al., 2023a; Mäkinen et al., 2020) there are similarities and differences between Finnish and Italian children in their pragmatic performance. The current study extends previous findings showing that there is a similar developmental trend in pragmatic comprehension among the two groups of children, despite the Finnish children performing slightly better across the age groups. A closer look at the overall data suggests the presence of similarities in the youngest groups, with a pattern of performance that seems to differentiate more starting around 6 years of age. In addition, the current study showed that by using structured test materials that focus on contextual processing, it is possible to explore the development of complex communication abilities in a relevant and effective way and in different cultural and linguistic contexts.

Having found that a similar developmental pattern can be detected among Italian and Finnish children, it can be suggested that the underlying abilities required for pragmatic comprehension follow the same acquisition pattern, and this might reflect a universal sequence of developmental stages. On the other hand, some specificity in communicative features can be detected, being culture-specific or language-specific (see Küntay et al., 2014). As suggested by Sperber and Wilson (1995), pragmatic comprehension is founded on upholding the concept of relevance in specific communicative situations which is a reflection of human cognition. Within this common pattern, there can be differences in pragmatic performance which can reflect individual experiences, abilities as well as cultural and linguistic contexts.

In this study, we found that Finnish children in all age groups had higher performance scores on the Pragma test. However, the difference in performance was less pronounced at the age of four. While the difference in performance increases at the age of five, there is a marked change in the difference at the age of six, which persists until the age of eight. Looking at contextual comprehension from a cross-cultural perspective and by age groups is important for understanding different stages of development. This has particular relevance for early education and clinical contexts, as it may contribute to shedding light on similarities and specificities in dealing with communicative challenges in different everyday contexts.

When looking at the different question types we interestingly found that the Finnish and Italian children performed similarly with regard to questions requiring recognition of feelings depicted in different contexts. This was quite unexpected in light of previous evidence where Italian children were found to express feelings more readily than Finnish children. This was the case, for example, in a comparative study on narrative production, where Finnish children were less likely than Italian children to explicitly mention the story characters’ emotional reactions (Mäkinen et al., 2020). It is nevertheless worth noting that expression and recognition of feelings may be considered quite different processes, differently impacted by cultural habits, which may explain the disparity among these results. The present study, indeed, suggests that the Finnish style generally considered as more introverted (Lehtonen and Sajavaara, 1985; Sallinen-Kuparinen, 1986) when compared to the Italian one, does not result in a lower capacity to tune to others’ feelings and correctly interpret them.

The most significant differences between the Finnish and Italian children on the different question types were found with questions requiring contextual processing without Theory of Mind (*ConNoToM*) and understanding relevant use of language (*RelUseLang*). In these question types the Finnish children performed better than the Italian children. About the *ConNoToM* questions, while the developmental pattern is similar, the Finnish children performed better than the Italian children at all ages. For the *RelUseLang* questions all the children performed similarly in the age groups four and five. However, from age six onwards, the Finnish children performed significantly better than the Italian children on the questions requiring understanding of relevant use of language. This set of results, showing different developmental trajectories across age groups might reflect the influence of multiple environmental and contextual factors that were not directly assessed in the present study. These may include differences in educational experiences, family routines, opportunities for social interactions, social support, structure and quality of the school system. Therefore, the following considerations should be regarded as plausible interpretations rather than conclusions supported by the present data. Particularly interesting in this regard is the change in the trend that is detectable in our results starting at the age of six. In both countries, this age corresponds to an important educational transition, although children enter different educational settings (mandatory pre-primary education in Finland and the first year of primary school in Italy). It is therefore possible that differences in educational experiences contribute to the observed developmental trajectories. However, because educational characteristics were not measured in the present study, this interpretation remains speculative and warrants direct investigation in future research.

Specifically, the questions belonging to the *RelUseLang* type assess children’s ability to understand that there are different ways to use language depending on the context and imply the ability to choose how to express a concept or a request adhering to a particular situation and interlocutor (see Loukusa, 2019). This ability, more than others, is likely to be shaped by experience and (implicit) teaching. Compared with other pragmatic skills, this competence may be particularly sensitive to accumulated communicative experience, explicit instruction, and everyday language exposure. Accordingly, differences in educational practices or broader communicative environments could represent one of several possible explanations for the observed cross-cultural differences, although the present findings do not allow us to determine their relative contribution.

In this regard, it is also relevant noting that Finland has received considerable attention attention in recent years for the reported high quality of its school system, testified by a persistent leadership in the ranking provided by the Program for International Student Assessment (i.e., PISA) (see Organization for Economic Cooperation and Development, 2017; Organization for Economic Cooperation and Development, 2023 for a detailed overview of the ranking worldwide in the past years). Although this contextual information may offer a useful perspective for interpreting the present findings, it should not be considered evidence that educational quality accounts for the differences observed in this study. Establishing such a relationship would require studies specifically designed to measure educational experiences and their association with pragmatic development. Finally, regarding the false belief questions, while the Finnish children outperformed the Italian children until age six, from age seven onwards both groups performed near ceiling, suggesting that cross-cultural differences become negligible once this component of pragmatic competence is fully acquired.

As previously mentioned, we did not limit the investigation to pragmatic comprehension, but we also focused on children’s ability to reason about their answer, as an indirect index of metacognitive ability. To the best of our knowledge such aspect has received very little attention from a cross-cultural perspective. An exception is the study of Kim et al. (2020), detecting no difference between Japanese and German children in a metacognitive task. As for this aspect, in the present study we found that when the children were required to explain how they arrived at their correct answers, the Finnish children had higher scores and the biggest divergence was evident at age five. This result indicates that Finnish children draw upon their metacognitive and linguistic skills earlier when required to explain answers than the Italian children, and then the ability appears to stabilize. From age six onwards, indeed, the difference becomes less pronounced, the gap narrowing towards age eight. As stated above, it seems reasonable to explain some of the results whose pattern changes as a function of age by referring to a variety of cultural and societal factors, among which the school system might play an important role. On the other hand, it is also possible to assume that cross-culturally divergent developmental patterns may be expected when it comes to communicative ability: some differences might be, indeed, detectable at early stages and then flatten out later on. This latter phenomenon was detected by Mäkinen et al. (2020) showing that some of the differences (e.g., number of words produced in a narrative task) detected when comparing Finnish and Italian children at 4 years of age disappeared when comparing children of 8 years of age on the same variable. It is also noteworthy to consider that although the explanation tasks were scored according to standardized criteria focusing on the adequacy of children’s reasoning rather than on the linguistic form of their responses, we cannot exclude that broader linguistic-cultural conventions regarding verbal elaboration and explanation may have influenced children’s performance. Future studies should further investigate this issue.

Finally, for explorative purposes, we examined possible gender differences in both pragmatic comprehension and in the ability to reason on one own’s answers. Overall, the results indicate that girls outperformed boys on both measures. However, inspection of the two cultural groups separately suggested a larger disparity between boys and girls in the Finnish sample, whereas among Italian children the scores of boys and girls were basically overlapping. Although none of the interactions involving gender reached statistical significance (i.e., Nationality × Gender and Nationality × Age × Gender interactions), these null findings should be interpreted with caution. While the overall sample size was adequate to detect the main effects reported in this study, the factorial design (2 nationalities × 2 genders × 5 age groups) resulted in relatively small numbers of participants within each subgroup, limiting statistical power to detect higher-order interactions. Consequently, subtle age- or culture-related modulation of gender differences cannot be ruled out and should be investigated in future studies with larger samples. Taken together, the present findings add to the mixed literature on gender differences in language and communication skills. Previous studies had reported girls outperforming boys in engaging in spontaneous conversations (e.g., Bauer et al., 2002), or in storytelling skills (Fey et al., 2004); on the other side, other studies failed to detect any difference in communication or social skills based on gender (e.g., Collins et al., 2014; Helland et al., 2009; Law et al., 2014; see also Etchell et al., 2018 for a review). A similar pattern of results seems to apply to metacognitive ability (e.g., Xue et al., 2024). The number of evidence on this topic decreases even more when considering also cross-cultural variability. To the best of our knowledge, only a previous study (Gabbatore et al., 2023a) investigated gender differences in Finnish and Italian children of preschool- and school-aged, using the same tool for communicative assessment, i.e., CCC-2 (Bishop, 2003). Consistent with the present findings, girls showed more strengths than boys in some of the communicative aspects investigated (e.g., coherence, appropriate initiation) with a similar pattern of results in the two samples, as evident from the non-significant interaction Nationality × Gender, also in that case.

Large scale cross-cultural studies, such as this, are important for gaining understanding of developmental patterns as well as similarities and differences in development. The cross-cultural dimension of this study provides an opportunity to explore universal and language- and culture-specific factors in development. The present data, in this regard, seem to indicate the existence of a common matrix when it comes to pragmatic development within the Finnish and Italian cultural contexts, as suggested by the similar developmental trends of the children at the Pragma scores. Nevertheless, such a common starting point, detectable while observing a similar pattern of performance in the youngest age groups, seems to lose its universality as soon as we move towards the older groups, with cultural factors appearing to play a progressively greater role.

More broadly, we would like to point out that the present findings should not be interpreted as evidence of a unitary “cultural effect.” Rather, culture could be better conceptualized as a multidimensional developmental context rather than as a single causal variable (see Bronfenbrenner and Morris, 2006). From this perspective, the differences (and similarities) observed between Finnish and Italian children are more appropriately interpreted as reflecting two distinct linguistic-cultural contexts, which may encompass a combination of interacting influences related to language, communicative practices, educational experiences, family interaction patterns, and socioeconomic conditions. The present study was not designed to disentangle the relative contribution of these individual components; therefore, nationality, here used as a grouping variable for methodological reasons, should be regarded as a methodological proxy for this broader developmental context rather than as a direct causal explanation of the observed differences.

Besides its merits, a limitation of the present study is that cognitive and linguistic abilities (e.g., vocabulary, structural language, working memory, and executive functions) were not directly assessed. Consequently, although all participants met the inclusion criteria and showed typical developmental histories according to parent report, we cannot determine the extent to which individual or cross-cultural differences in these underlying abilities contributed to the observed differences in pragmatic performance. Cognitive and pragmatic skills are, indeed, strongly related along development (see, e.g., Matthews et al., 2018; Pompei et al., 2025) and future studies should integrate standardized measures of cognitive and linguistic functioning with pragmatic assessment to better disentangle these contributions.

A unique feature of this study is the use of a structured tool, the Pragma Test, validated for two cultural contexts, which provides comparable data for exploring complex pragmatic processing. The translation and adaptation process allowing such a comparison (see Gabbatore et al., 2021) is far from easy and, surely, a candidate explanation we cannot rule out for the observed Finnish advantage is the fact that the tool was originally developed in the Finnish cultural context, despite the careful set of procedures followed to guarantee the validity, reliability and robustness of the tool (see Gabbatore et al., 2021; Loukusa et al., 2017). In any case, making such a comparison possible is not insignificant given that pragmatic performance is often studied in open communicative situations which do not lend themselves easily for objective cross-linguistic comparison. To gain a fuller understanding of children’s pragmatic performance it is desirable to integrate different methodologies and assessment tools, as for example experimental situation and parental report (Gabbatore et al., 2023a; see also Küntay et al., 2014). In the context of global mobility, physical and virtual, understanding how communication is situated in different cultural and linguistic contexts aids mutual understanding and global cohesion.

Despite the amount of work done, much still remains to be done, and several avenues for future research emerge from the present findings. We have here concentrated on pragmatic comprehension, but it would be equally interesting to gain further understanding of cultural factors also affecting pragmatic production. In addition, although socioeconomic status was not significantly associated with children’s pragmatic performance in the present sample, future studies should incorporate more comprehensive measures of socioeconomic status to further clarify the respective contributions of socioeconomic and cultural factors to pragmatic development. Moreover, it would be important to compare the language proficiency of children from different age groups and cultural contexts in various everyday situations to gain more precise information about when cultural influence becomes most pronounced. Also, it would be interesting to conduct longitudinal studies, which would allow further fine-grained analyses and interpretations. Finally, although the Italian version of the Pragma Test was developed from the original Finnish version through a rigorous process of translation, cross-cultural adaptation, and psychometric validation (Gabbatore et al., 2021), an additional step for future research might be to formally examine measurement invariance across the Finnish and Italian versions. Such analyses would provide further evidence that the instrument functions equivalently across languages and cultural contexts, thereby strengthening the interpretation of cross-cultural comparisons.

## Data Availability

The datasets presented in this article are not readily available because of privacy or ethical restrictions. Requests to access the datasets should be directed to Ilaria Gabbatore (ilaria.gabbatore@unito.it) and Soile Loukusa (soile.loukusa@comparte.fi).
